# Safe Use of Immune Checkpoint Inhibitors in Patients With Autoimmune Disease in a Community Hospital Setting: A Report of Two Cases

**DOI:** 10.7759/cureus.81877

**Published:** 2025-04-08

**Authors:** Vivek D Shah, Surbhi Bansil, Jarred P Reed

**Affiliations:** 1 Hematology and Medical Oncology, Olive View University of California Los Angeles Medical Center, Los Angeles, USA; 2 Internal Medicine, Olive View University of California Los Angeles Medical Center, Los Angeles, USA; 3 Oncology, Olive View University of California Los Angeles Medical Center, Los Angeles, USA

**Keywords:** autoimmune disease, clinical trial inclusion, immune checkpoint inhibitor, immune related adverse events, rheumatologic disease

## Abstract

Immune checkpoint inhibitors (ICIs) are fundamental to the management of hematologic and solid organ malignancies, and indications for their use are rapidly increasing. Patients with preexisting autoimmune diseases are often excluded from clinical trials involving ICIs due to the risk of exacerbating the underlying autoimmune condition. The lack of trial-level safety data limits the use of these therapies in real-world patients with autoimmune disease. We present a case series of two patients with preexisting autoimmune diseases who received immunotherapy for their malignancies, with manageable toxicities and desirable outcomes from an oncologic standpoint. We aim to contribute to the growing body of evidence suggesting that ICIs can be safely offered to selected patients with autoimmune diseases.

## Introduction

Tumor-infiltrating lymphocytes (TILs) within the tumor microenvironment help target and eliminate tumor cells via the interaction between T-cell receptors and foreign antigens presented in the major histocompatibility complex of tumor cells. T cells recognize these antigens as foreign, leading to the activation of immune responses. It is now known that tumor cells can evade TILs through the expression of inhibitory immunoreceptors, or “immune checkpoints,” such as CTLA-4, LAG-3, and PDL-1, which reduce TIL activation [1,2].

In 2011, immune checkpoint inhibitors (ICIs) were introduced as a new category of therapies aimed at targeting immune checkpoint pathways to mitigate this tumor response [3,4]. The first FDA-approved ICI was ipilimumab, after data showed that its use improved overall survival in patients with advanced melanoma [5]. Since then, the roles and variety of ICIs have broadened exponentially, and ICIs now have a role in neoadjuvant, adjuvant, and metastatic settings, often with remarkable survival benefits.

Patients with autoimmune diseases who receive ICIs are at increased risk of immune-related adverse events (IRAEs) and flares of their autoimmune disease due to immune system activation. One meta-analysis found that, of 123 patients who received immunosuppressive therapies for preexisting autoimmune diseases at the time of initiating ICIs, 75% suffered from an acute flare of their autoimmune disease, IRAEs, or both [6].

Patients with autoimmune diseases generally have a higher incidence of several malignancies, and certain autoimmune conditions are associated with a markedly increased risk. Most notably, patients with polymyositis and dermatomyositis have a standardized incidence ratio (SIR) of 3.0, with ovarian, lung, and gastrointestinal tract cancers being the most common [7]. A retrospective analysis of individuals with rheumatoid arthritis showed an SIR of 2 for lymphoma and 1.63 for lung cancer. The mechanism for this is postulated to be related to a chronic, dysregulated inflammatory state, which can result in clonal lymphocyte proliferation and a higher risk of developing a malignant clone [8]. There is also a suggestion that, in some patients, a preexisting autoimmune condition may actually be a paraneoplastic syndrome and, therefore, the presenting sign of a malignancy [7]. In such cases, management of the malignancy may be the most effective treatment for the autoimmune symptoms.

Given the efficacy of ICIs, it is imperative to establish a protocol to safely administer these drugs to patients who are at higher risk of morbid outcomes, particularly those with autoimmune diseases. While rates of autoimmune flares and de novo IRAEs may be higher in this population, we posit that, for many patients with autoimmune conditions, ICIs can be safely offered. We report a case series of two patients with active autoimmune disease who received ICIs after thorough discussions regarding the potential risk of IRAEs. Through multidisciplinary collaboration and close monitoring, these patients achieved favorable responses to therapy without significant adverse outcomes.

## Case presentation

Case 1

A 68-year-old male with a 50 pack-year smoking history presented with unintentional weight loss of 11 kg over several months, diffuse symmetric arthralgias, and early satiety. The patient had not previously seen a healthcare provider on a consistent basis, so his baseline health was unclear. He was unaware of his family history, as they were estranged. He was a retired war veteran who served in Vietnam.

Computed tomography (CT) scans of the chest, abdomen, and pelvis revealed a 3.7-cm spiculated right apical lung mass, an adjacent 1.3-cm right upper lobe mass, extensive lymphadenopathy (mediastinal, paratracheal, axillary, and retroperitoneal chains), a 1.5-cm adrenal mass, and a 2.4-cm peritoneal nodule. A biopsy of the right lung mass revealed poorly differentiated adenocarcinoma with a tumor mutational burden of 50 mutations/Mb, without any actionable genetic alterations. The patient was also evaluated by Rheumatology for his history of extensive arthralgias. Physical examination was notable for a swan neck deformity of his right third digit and flexion contractures of both elbows. Laboratory data showed an elevated rheumatoid factor at 3063 U/mL and elevated anti-cyclic citrullinated peptide at 101 EU/mL. Together, his clinical manifestations met the criteria for a diagnosis of rheumatoid arthritis, and he subsequently started immunosuppressive therapy, including corticosteroids at 1 mg/kg/day, which were tapered off over six weeks as tolerated.

**Figure 1:**
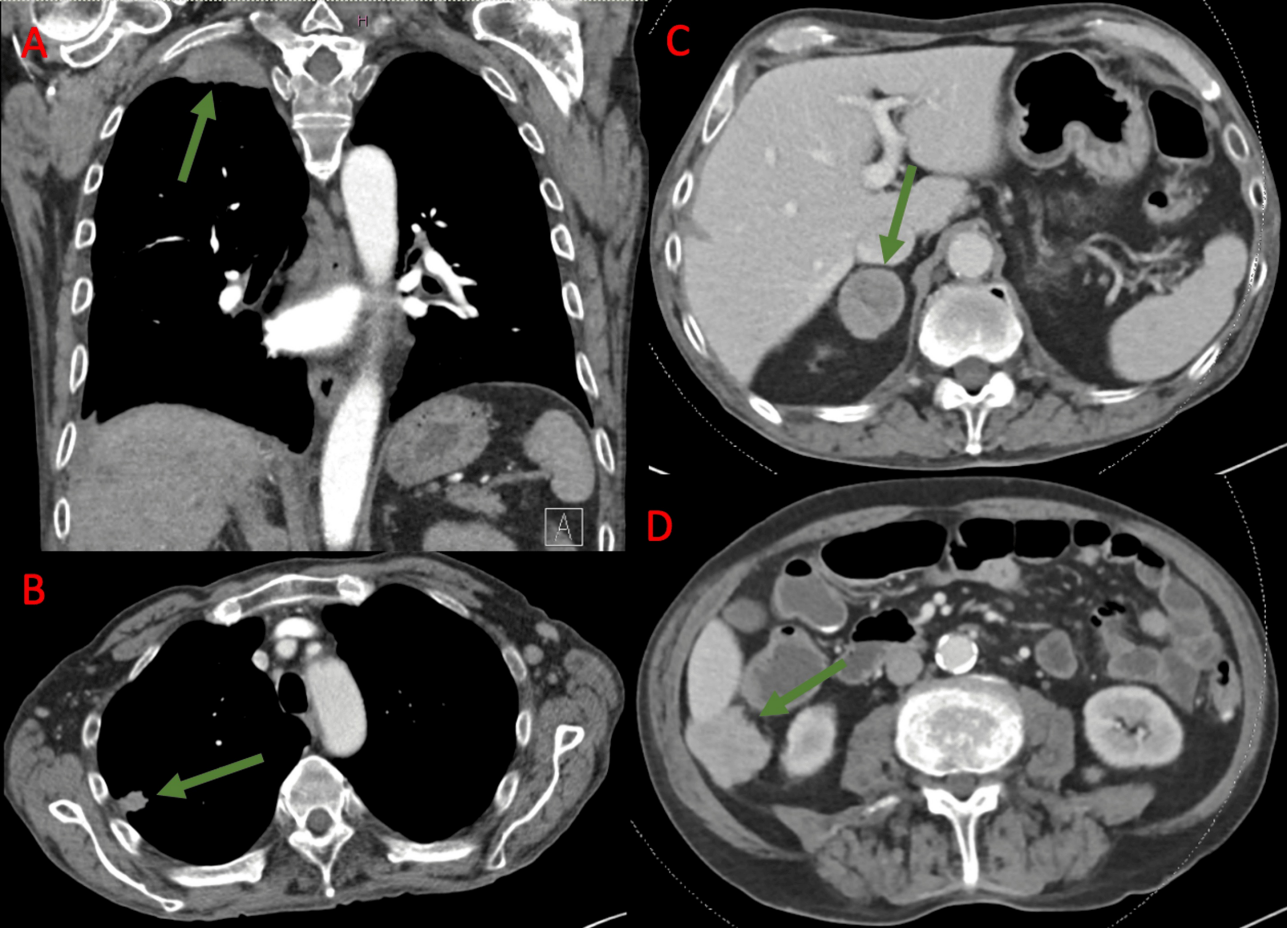
Target lesions prior to initiation of immune checkpoint inhibitor therapy, demonstrated on computed tomography. (A) Apical lung mass measuring 4.6 cm. (B) Pulmonary nodule measuring 1.3 cm. (C) Adrenal nodule measuring 3.7 cm. (D) Right peritoneal nodule measuring 3.5 cm. The green arrow points to the lesion of interest in each image.

After discussion between Rheumatology and Oncology, it was determined that it would be best to trial cytotoxic chemotherapy as first-line treatment for this patient’s lung adenocarcinoma. The patient was treated with carboplatin (AUC 2) and paclitaxel 100 mg/m² weekly, with the paclitaxel subsequently dose-reduced by 20% to 80 mg/m² at cycle 2 due to myelosuppression. After 10 weeks of therapy, repeat imaging showed that the lung mass had increased in size to 4.6 cm, the right pulmonary nodule measured 1.3 cm, the adrenal nodule had grown to 3.7 cm, and the right peritoneal nodule to 3.5 cm (Figure 1).

Because the patient’s cancer rapidly progressed on first-line chemotherapy, Oncology and Rheumatology discussed the potential use of immune checkpoint inhibitor (ICI) therapy. Oncology suggested that ICI might offer a durable response due to the high tumor mutational burden. Rheumatology agreed to closely monitor the patient for signs of rheumatoid arthritis flare and to titrate immunosuppressive therapies as needed. Pembrolizumab was initiated at a standard dose of 200 mg every three weeks. Concurrently, Rheumatology started hydroxychloroquine (200 mg daily) and methotrexate (15 mg weekly), and continued low-dose prednisone (10 mg daily) to optimize treatment of the patient’s rheumatoid arthritis.

**Figure 2:**
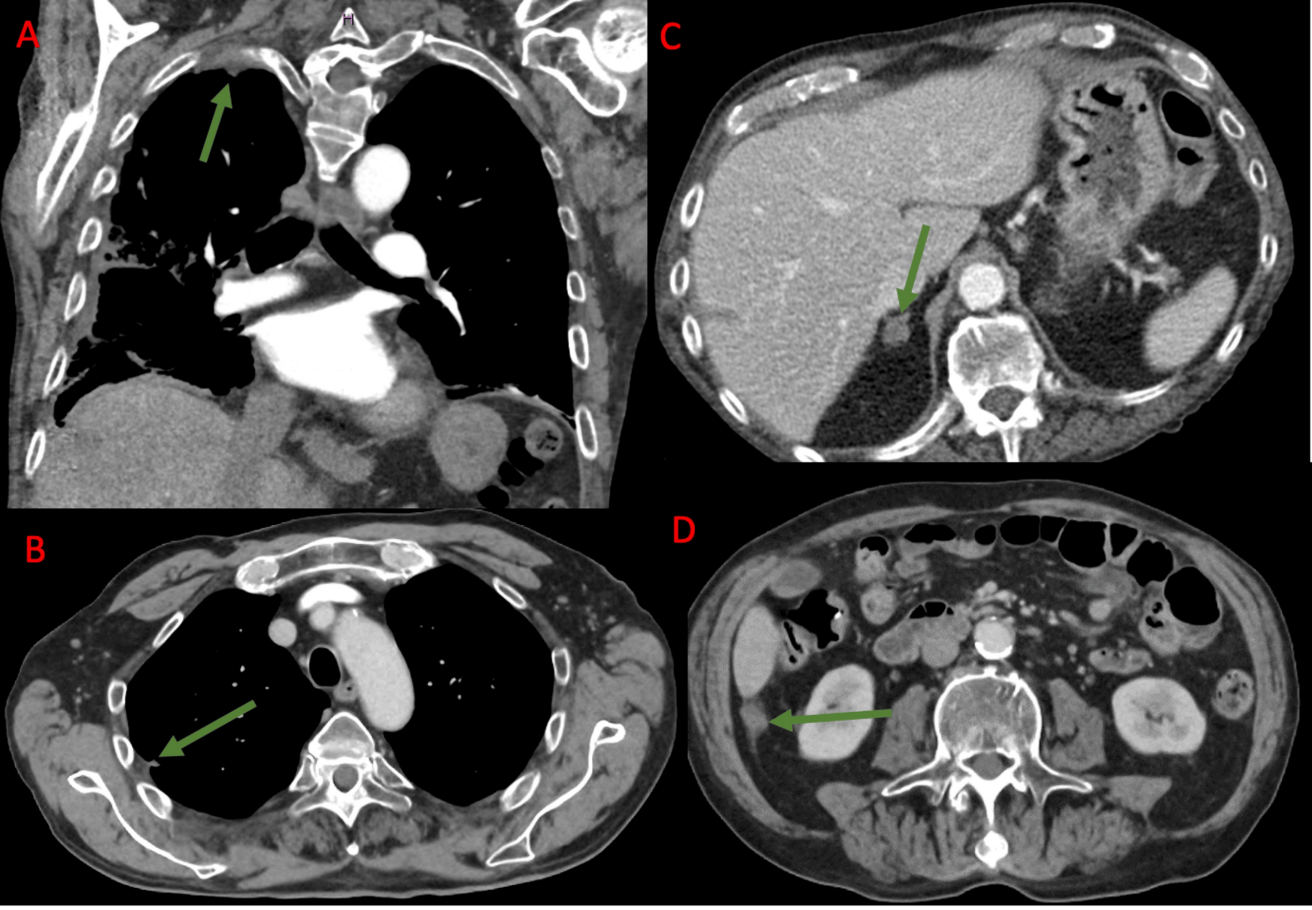
Target lesions post-ICI therapy demonstrated on computed tomography. (A) Right apical mass now measuring 3.4 cm. (B) Right pulmonary nodule now measuring 0.7 cm. (C) Adrenal nodule now measuring 1.2 cm. (D) R peritoneal nodule now measuring 1.3 cm. The green arrow points to the lesion of interest in each image. ICI: immune checkpoint inhibitors.

After six cycles of pembrolizumab, treatment was held due to grade 2 diarrhea, suspected to be secondary to ICI-induced colitis. A steroid taper was initiated, and the patient’s symptoms resolved within one week. Imaging at that time revealed a significant response, with the right apical lung mass decreasing from 4.6 cm to 3.4 cm, the pulmonary nodule from 1.3 cm to 0.7 cm, the adrenal nodule from 3.7 cm to 1.2 cm, and the right peritoneal nodule from 3.5 cm to 1.3 cm (Figure 2). Unfortunately, at this time, the patient was hospitalized due to complications from widespread atherosclerotic disease, which included bowel and limb ischemia as documented on CT angiography. He died before further treatment could be administered.

Case 2

**Figure 3:**
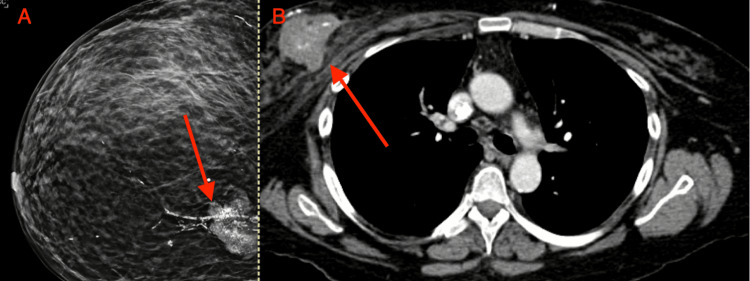
Imaging obtained prior to initiation of neoadjuvant therapy. A. Mammogram demonstrating a right upper quadrant breast mass with pleomorphic calcifications. B. View of right breast mass on CT scan. Red arrow points to the lesion of interest in each image.

A 42-year-old woman with a history of systemic lupus erythematosus (SLE), complicated by lupus nephritis and end-stage renal disease requiring intermittent hemodialysis, managed with hydroxychloroquine and prednisone, underwent a pre-transplant screening mammogram and was found to have a right breast mass measuring 4.1 × 3.9 × 2.6 cm, with calcifications within the mass extending up to 8 cm (Figure 3). Further imaging and biopsy revealed poorly differentiated triple-negative invasive ductal carcinoma with a Ki-67 of 90%. The case was discussed at a tumor board, and a plan was made for neoadjuvant chemotherapy followed by surgery. Based on this patient’s triple-negative cancer histology, the standard of care is to provide chemotherapy and immune checkpoint inhibitor (ICI) therapy preoperatively, based on the KEYNOTE-522 study [9]. However, the patient wished to avoid the risk of an ICI-associated SLE flare, so she was given standard chemotherapy with doxorubicin 45 mg/m² and cyclophosphamide 450 mg/m² (both dose-reduced by 25% to account for ESRD on dialysis) every two weeks for a total of four cycles, followed by 12 weekly doses of paclitaxel 80 mg/m² [10]. Following neoadjuvant therapy, she underwent a simple right breast mastectomy with sentinel lymph node dissection, which showed residual disease measuring 4.5 cm in greatest dimension and a closest margin of 2.1 cm, with no lymph node involvement. 

**Figure 4:**
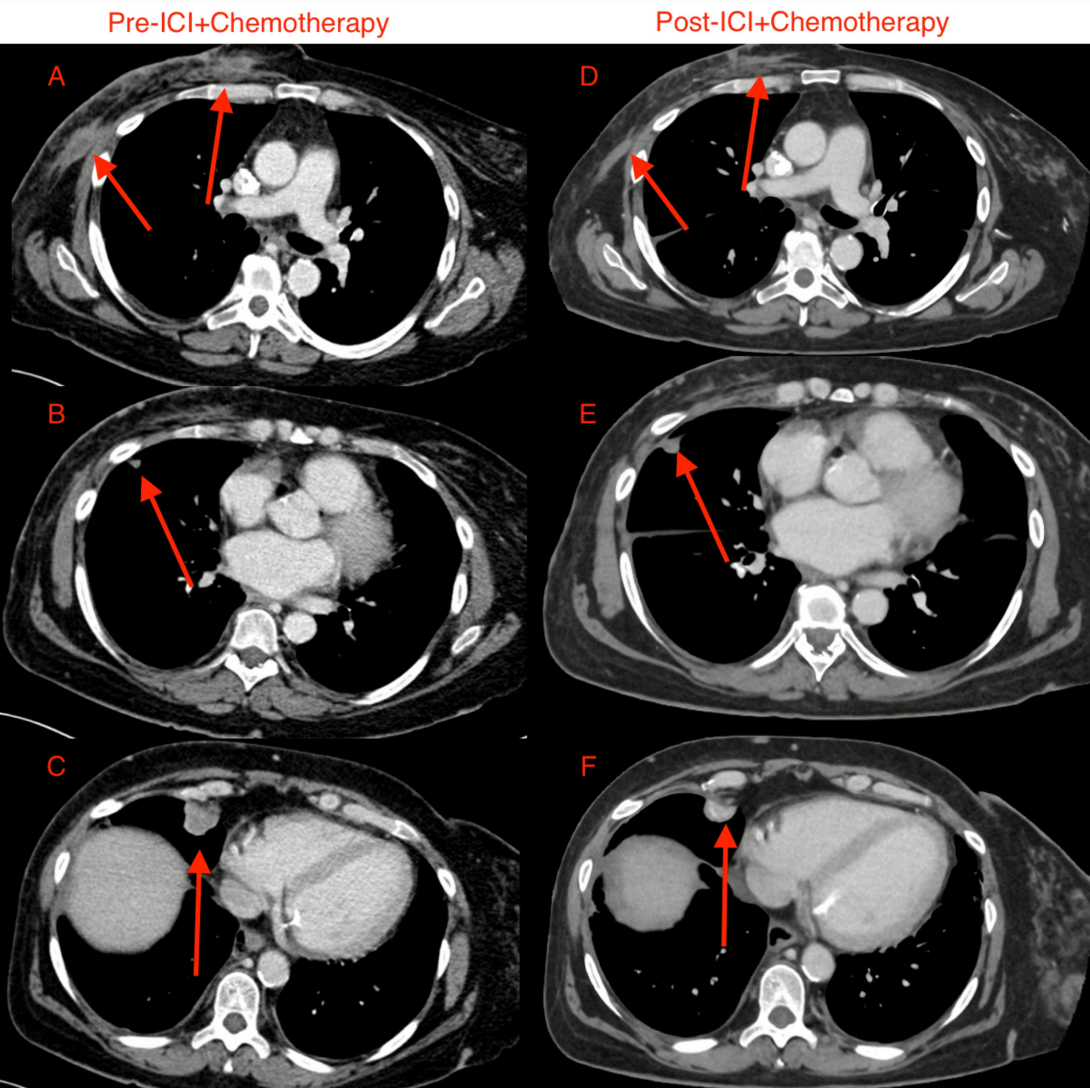
Metastatic foci seen on CT scan prior to and after initiation of ICI + Chemotherapy. (A) Two right chest wall lesions prior to treatment. (B) Right middle lobe juxta-fissural pulmonary nodule prior to treatment. (C) Right middle lobe pulmonary nodule prior to treatment. (D) Two right chest wall lesions after treatment. (E) Right middle lobe juxta-fissural pulmonary nodule after treatment. (F) Right middle lobe pulmonary nodule after treatment. The red arrow points to the lesion of interest in each image. ICT: immune checkpoint inhibitors.

Within two months of surgery, prior to the initiation of adjuvant radiation therapy, repeat imaging revealed metastatic recurrence to the lungs and chest wall. The new findings included two peripherally enhancing lesions of the right chest wall measuring 2.4 × 1.8 × 3.1 cm and 3.0 × 1.7 × 2.6 cm, a solitary right middle lobe pulmonary nodule measuring 2.1 cm, a 6-mm right middle lobe juxta-fissural nodule, and nodular right pleural thickening (Figure 4). A repeat biopsy confirmed triple-negative breast cancer with a PD-L1 CPS of 10 and no actionable mutations. The patient discussed with both Oncology and Rheumatology the risks of SLE exacerbations and the potential benefits of disease control for her seemingly rapidly progressive breast cancer with the administration of ICI. Based on this discussion, she was initiated on weekly carboplatin (AUC 2), gemcitabine 800 mg/m², and pembrolizumab 200 mg, with close monitoring of her well-controlled SLE [11].

The patient received eight cycles of treatment with clinically and radiographically stable disease (Figure 4). She experienced no immune-related adverse events during her treatment with pembrolizumab.

## Discussion

We have described two cases of patients with preexisting autoimmune diseases who received immunotherapy for their malignancies, with manageable toxicities and desirable outcomes from an oncologic standpoint. Our patient with advanced lung adenocarcinoma survived over nine months with ICI, of which the last four months were off therapy, suggesting the possibility of a durable response. Our patient with recurrent metastatic triple-negative breast cancer had limited treatment options; however, with ICI, she achieved stable disease for six months.

Our patient with TNBC received an ICI in the metastatic rather than neoadjuvant setting, by which time her disease was incurable. This raises the question of whether patients with autoimmune disease, in efforts to cure aggressive early-stage disease and offer long-term survival, should be considered candidates for ICIs. Specifically, per KEYNOTE-522, in early-stage disease, 36-month overall survival was 84.5% in the treatment arm (pembrolizumab + chemotherapy) vs. 76.8% in the chemotherapy-alone arm. More recently, additional data were published showing that KEYNOTE-522 met the pre-specified endpoint of overall survival [9,12].

To date, prospective randomized trials studying the use of ICIs have excluded patients with autoimmune disease. Given the high prevalence of autoimmune disease in the cancer population, in recent years, multiple groups have attempted to address this unmet need via retrospective studies examining the safety and outcomes of ICI use in these patients. For example, a large single-center retrospective study included 147 patients with preexisting autoimmune disease, with 18 different malignancies represented [12]. Autoimmune disease flare and/or IRAE in the autoimmune disease patient cohort occurred in 59.1%; however, 81% of autoimmune disease flares and 76% of IRAEs were grades 1-2, indicating that, in most patients, toxicity was mild. It should be noted that individuals with their autoimmune disease under control had much lower rates of flare (60% vs. 20%). Of note, and in accordance with data from clinical trials, those who suffered IRAEs had higher response rates (42.5% vs. 8.3% in patients with no IRAEs). Unfortunately, in patients with active autoimmune disease, though treatment response was similar, overall survival was poorer, possibly due to poorer baseline health and the need for more intensive immunosuppressive therapy [13].

Another matched comparative retrospective study found that, in comparing 87 patients with preexisting rheumatoid arthritis (RA) with 203 patients without RA, the RA group had higher rates of IRAEs of any grade versus matched control comparators (61% vs. 49%), though this difference was not statistically significant. In the RA group, 48% suffered disease flare, whereas 7% of matched controls experienced inflammatory arthritis, suggesting that most IRAEs seen in patients with RA were attributable to flare of their existing conditions. This same study found similar rates of grade 5 toxicity and mortality in both groups [14]. A single-institution study that examined the incidence of autoimmune disease flare and IRAEs in patients with preexisting autoimmune diseases and advanced solid malignancies treated with ICIs found that, among 40 patients, the rate of high-grade IRAEs was 20% in the autoimmune disease group versus 5% in the general cancer population [15].

These and other retrospective studies have shown that ICIs appear to be effective and safe in carefully selected patients with stable autoimmune disease. Fortunately, there are now ongoing prospective studies investigating the toxicity profiles in patients with concomitant autoimmune disease and metastatic malignancies on ICI. The AIM-NIVO study is an ongoing Phase Ib study aimed at determining the occurrence of ICI-related toxicities and adverse events, as well as overall survival and progression-free outcomes, among patients with preexisting autoimmune diseases treated with the anti-PD-1 ICI nivolumab for advanced malignancies [16]. Such studies may help expand access to ICIs among patients with autoimmune diseases.

## Conclusions

Now that more data are becoming available, specifically pertaining to the use of ICIs and the expanding indications for their use in the treatment of various malignancies, it is imperative that oncologists do not broadly withhold such treatment from patients with autoimmune conditions. Based on available evidence and our personal experience as described in this report, we recommend a multidisciplinary approach to determine whether patients with autoimmune conditions who would otherwise be eligible for ICI therapy are appropriate candidates for initiation of treatment with close monitoring for IRAEs or disease flare. In particular, patients with aggressive malignancies, high response rates to ICIs, and autoimmune diseases that are well controlled may be optimal candidates for this approach. Ongoing close collaboration with Rheumatology is critical to optimize control of autoimmune disease and monitor for toxicity while patients are on ICI therapy. Lastly, education of patients, caregivers, nursing staff, and other providers can help ensure that toxicities are recognized and addressed promptly, to prevent morbidity and mortality while allowing the opportunity for robust responses to treatment.
